# Long-term mindfulness training is associated with reliable differences in resting respiration rate

**DOI:** 10.1038/srep27533

**Published:** 2016-06-07

**Authors:** Joseph Wielgosz, Brianna S. Schuyler, Antoine Lutz, Richard J. Davidson

**Affiliations:** 1 Center for Healthy Minds, University of Wisconsin–Madison, Madison, WI, 53705, USA; 2 Waisman Laboratory for Brain Imaging and Behavior, University of Wisconsin–Madison, Madison, WI, 53705, USA; 3 Department of Psychology, University of Wisconsin–Madison, Madison, WI, 53706, USA; 4 Lyon Neuroscience Research Center INSERM U1028, CNRS UMR5292, Lyon 1 University, Lyon, 69500, France

## Abstract

Respiration rate is known to correlate with aspects of psychological well-being, and attention to respiration is a central component of mindfulness meditation training. Both traditional contemplative systems and recent empirical evidence support an association between formal mindfulness practice and decreased respiration rate. However, the question of whether long-term mindfulness training is associated with stable, generalized changes in respiration has yet to be directly investigated. We analyzed respiration patterns across multiple time points, separated by two months or more, in a group of long-term mindfulness meditation practitioners (LTMs, n = 31) and a matched group of non-meditators (Controls, n = 38). On average, LTMs showed slower baseline respiration rate (RR) than Controls. Among LTMs, greater practice experience was associated with slower RR, independently of age and gender. Furthermore, this association was specific to intensive retreat practice, and was not seen for routine daily practice. Full days of meditation practice did not produce detectable changes in baseline RR, suggesting distal rather than immediate effects. All effects were independent of physiological characteristics including height, weight, body-mass index and waist-hip ratio. We discuss implications for continued study of the long-term effects of mindfulness training on health and well-being.

Respiration rate is a physiological correlate of psychological well-being. In particular, chronic rapid, irregular breathing patterns are associated with increased anxiety^1^, as well as clinical pain and anxiety disorders^2^. Conversely, controlled breathing, especially deep, slow breathing, is established as an effective short-term intervention in behavioral health settings^3^,^4^, and has been shown to reduce autonomic reactivity, negative mood, and pain-related distress^5^.

Attention to breathing is a foundational component of mindfulness training, both in contemporary forms^6^ and the Buddhist traditions from which they are derived^7^. These traditions furthermore recognize an association between mindful attention to the breath and calming of both body and mind^7^,^8^.

In the past, small studies of experienced meditators have reported decreased respiration rate during periods of formal mindfulness-related meditation practice^9^,^10^,^11^. In at least one such study, decrease in respiration rate during meditation, relative to baseline, was observed to correlate positively with measures of practice experience^12^, suggesting that greater expertise may amplify this effect. Such effects have also been noted in recent studies of novice meditators after short-term instruction and training^13^,^14^,^15^. These studies support the conclusion that mindfulness training leads to slower respiration rate during formal practice itself. However, reduced respiration rate has considerably more relevance to psychological well-being if it is a sustained and generalized change that extends beyond formal practice. Results to date have been primarily limited to observation of formal meditation practice sessions, or instructed tasks, at a single laboratory visit. Additionally, most studies have relied on either novice mindfulness practitioners, or very small samples of experienced practitioners.

Here, we extend current knowledge by investigating whether long-term mindfulness training is reliably associated with individual differences in baseline respiration rate, measured at rest. Using a larger sample than in previous studies, we analyzed respiration recordings collected from both long-term meditators (LTM) and a matched control group (Controls), during uninstructed periods at three laboratory sessions spaced, on average, 4.5 months apart. For LTMs, we used detailed meditation practice histories to assess the relationship between respiration rate at rest and lifetime practice experience. Additionally, prior to some of the laboratory sessions, LTMs completed a full day of meditation practice in a controlled environment.

Based on prior observations of formal practice, and of the effects of attention to respiratory sensations, as reviewed above, we hypothesized that long-term meditators would exhibit slower baseline respiration rates than non-meditators, and that greater lifetime practice experience would be associated with slower respiration rate at rest, across the three sessions. We tested for proximal effects of practice in long-term meditators by comparing sessions which followed a day of practice to those which did not. Finally, we explored the effects of specific forms of training by conducting separate analyses of intensive retreat practice and daily, short-duration practice experience among long-term meditators.

## Results

To establish reliability of the primary outcome measure, baseline respiration rate (RR), we calculated intra-class correlation^16^, type 1, separately for the LTM and Control groups, and for the sample as a whole. RR showed good reliability across sessions in both groups, appearing slightly higher in LTMs: for LTMs, ICC(1) = 0.85, 95% CI: (0.75, 0.93); for Controls, ICC(1) = 0.64, 95% CI: (0.43, 0.80); overall, ICC(1) = 0.77, 95% CI: (0.65, 0.85).

To represent the repeated-measures design, analyses of RR and practice experience were conducted using maximum-likelihood linear mixed effects models. Two main sets of analyses were conducted: first, to compare meditators with non-meditators, and second, to examine effects of practice in the LTM group. To test for immediate effects, RR was regressed on Day of Practice (Open Monitoring, Loving Kindness, or None). To examine relationships with lifetime practice experience, Total, Daily and Retreat practice hours were then added to this model.

**Figure 1:**
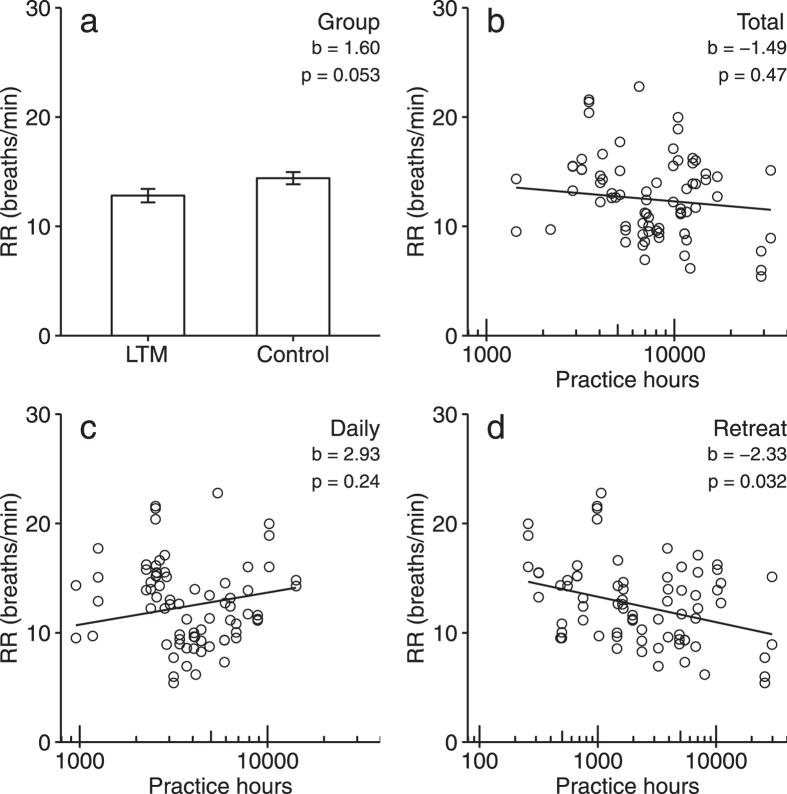
Mixed-effects models of relationships between meditation experience, and baseline respiration rate (RR), measured on three occasions per participant. Group differences (**a**) between Long-Term Meditators (LTM) and non-meditator Controls; error bars ± 1SE. Predicted relationships between RR and total lifetime practice experience (hours, log-transformed) for (**b**) total, (**c**) daily, and (**d**) intensive retreat practice, controlled for age and gender. RR: breaths / minute during 360 s of uninstructed rest.

To compare meditators with non-meditators, we first constructed a base model regressing RR on Age and Gender in the combined sample. No significant relationships were observed for either Age, t(68) = −0.82, p = 0.41, or Gender, t(67) = −0.83, p = 0.41. We next added Group to the base model. This model revealed a near-significant difference between groups, such that average RR was slower in the LTM group by 1.6 breath/min, t(64) = 1.97, p = 0.053 (see Fig. 1a). The effect of Group on RR did reach significance (p < 0.05) when modeled on its own, or when controlling for either Age or Gender alone. No difference in baseline RR was observed for between sessions which followed a Day of Leisure and those which did not, t(28) = 0.07, p = 0.94.

To investigate effects of practice, we first fit a base model regressing RR on Age and Gender in the LTM group. We observed a significant effect of Age, such that an increase of 1 years in age was associated with a decrease of 0.16 breaths/minute in RR, 95% CI: (0.04, 0.29), t(27) = −2.77, p = 0.01. There was no significant effect of Gender, t(27) = −1.4, p = 0.17. We extended the base model by adding Day of Practice predictors. This revealed no significant effects for either Open Monitoring, t(45) = 0.60, p = 0.55, or for Loving Kindness practice, t(45) = 0.37, p = 0.71. Next, we individually added predictors for log-transformed Retreat, Daily and Total lifetime practice hours (see Fig. 1b–d). A significant inverse relationship was observed between RR and Retreat hours, such that a doubling of Retreat hours was associated with a decrease of 0.70 breaths/min in RR, 95% CI: (0.07, 1.33), t(27) = −2.26, p = 0.032. No significant relationship was observed between RR and Daily hours, t(29) = 1.21, p = 0.24, or between RR and Total hours, t(27) = −0.73, p = 0.47.

To determine whether the main analyses were potentially explained by other physiological variables (height, weight, body mass index, waist-hip ratio, resting blood pressure, or resting heart rate), we added these variables individually to the base models. We found that none of these variables was a significant predictor of RR. Furthermore, their inclusion did not alter the direction or significance of the effects of either Group or Retreat hours. We did observe a significant difference between groups in weight, such that LTMs weighed 16.5 lbs less than Controls, 95% CI: (1.7, 31.3), t(69) = −2.21, p = 0.03. Accordingly, body-mass index (BMI) was also significantly lower by 2.43 for LTMs than Controls, t(68) = −2.73, p = 0.01. However, as noted above, neither weight nor BMI predicted RR on their own, nor, when included as secondary predictors, did they affect the association between Group and RR. No other significant differences were observed between the LTM and Control groups (all p > 0.10).

In summary, among the range of demographic and physiological characteristics variables we tested, respiration rate was uniquely associated with practice experience, and this association could not be accounted for by any other third variable.

## Discussion

We first confirmed that respiration rate (RR) was reliable in both LTM and non-meditator control groups. This finding supports the presence of stable individual patterns of respiration over long time intervals. RR is known to show test-retest reliability over shorter time spans^17^; however, our findings are novel in establishing that, among long-term meditation practitioners in particular, RR demonstrates reliability across sessions spanning months to years.

We next tested our prediction that sustained meditation practice would be associated with lower respiration rate. As a group, LTMs showed a small but potentially significant difference in baseline RR relative to controls. Additionally, across three sessions, we observed a robust inverse relationship between lifetime practice experience and baseline RR among LTMs. Previous studies have reported slowing of RR during formal meditation, or during instructed laboratory tasks, at a single time point. We extended this report by establishing that an association between greater practice experience and decreased RR is detectable during uninstructed rest, and across multiple temporally separated observations. In addition, we found that this relationship depends on practice time spent in long-term retreat, but not daily practice time. This novel finding is potentially important for understanding the mechanisms by which the direct effects of effortful formal practice can generalize to habitual characteristics. It also has implications for the design of mindfulness interventions involving formal meditation practice.

The differential effect on RR of intensive retreat time and daily practice hints at a possible mechanism underlying the relationship between long-term mindfulness training and lower baseline RR. A distinctive characteristic of intensive retreat practice is that it allows for extended, contiguous periods of physiological quiescence, with minimal exposure to complex environmental demands and extraneous stimuli. By contrast, during daily practice, external stimuli are more frequent, and the complexities and stressors of every day life are more immediate and cognitively accessible. A parallel to this contrast is apparent in a recent study relating RR with pain perceptions in a sample of meditation novices. A group who merely sat still in a meditation posture, with occasional instructions to take a deep breath (referred to as “sham meditation”), showed a correlation between reduction in pain ratings, and reduction in RR^13^. Meanwhile, participants who were given more specific mindfulness-based cognitive instructions showed reductions in pain ratings, but without an association with RR. Researchers suggest that this reflects a physiological relaxation mechanism of analgesia for the sham meditators, as opposed to a cognitively mediated mechanism for the novice meditation trainees. Exploring whether these patterns translate to intensive practice, we find that several other studies have confirmed that novice mindfulness trainees, who lack retreat experience, show pain reductions during meditation that are uncorrelated with observed reductions in RR^18^,^19^,^15^. By contrast, however, one study of pain processing in long-term practitioners – who typically have extensive retreat experience – found that reductions in RR during meditation practice *were* associated with reductions in pain ratings^11^. Together with the present findings, this suggests a model wherein retreat practice effects specific changes via extended periods of reduced physiological arousal, whereas daily practice sessions more closely resemble the effortful cognitive training engaged in by novices.

Taken as a whole, then, mindfulness training may involve a positive feedback loop whereby physiological calm increases resources available for cognitive training, while improved affect regulation feeds back into physiological state. One potential role for respiration in this process is as a means of consciously attending to and modulating physiological arousal. One recent study demonstrated that experienced meditators surpassed a control group on several measures of accuracy in detecting respiratory sensations^20^. Moreover, it has been observed that directing attention to respiration sensations results in decreased RR among novice mindfulness practitioners, but not in a control group^14^. Thus mindfulness training may lead to greater ability to both detect and respond to physiological arousal via sensing and modulating respiration patterns. This process would again likely best be learned and consolidated during intensive retreat practice, eventually leading to the generalized changes in habitual breathing patterns observed here.

Perhaps the most important question, which we are continuing to investigate, is how the reported differences in habitual respiration relate to other neural and physiological effects of long-term mindfulness training, including potential mental and physical health benefits. The differences in respiration rate observed here fall within the typical range of variation for healthy adults. However, respiration influences an extensive range of processes in the body, including autonomic tone, cardiac rhythms, and metabolism in both the brain and periphery, in ways that are only partially characterized. Meanwhile, interoceptive attention, and the neural systems which subserve it, are increasingly understood to play an important role in contemplative practice^21^, which in the case of mindfulness training, has led to growing interest in breath awareness^19^,^15^,^21^. In turn, recent studies by our group have uncovered evidence of alteration in a variety of neural and physiological processes among long-term mindfulness practitioners, including neural activity during sleep^22^,^23^, stress reactivity and inflammatory response^24^, and inflammation-related gene expression^25^. Respiration, therefore, is a valuable target of study in that it offers potential insight into the pathways by which contemplative practice may lead to this wide array of physiological changes.

Another question implied by the differentiation between retreat and daily practice is: what is the optimal distribution of daily practice versus intensive retreat time for producing beneficial long-term change? There is growing recognition of a need for greater sophistication and specificity in identifying active mechanisms within mindfulness training, especially relative to other interventions^26^,^27^,^28^,^29^. Currently, much translational research focuses on mindfulness interventions, such as Mindfulness-Based Stress Reduction^6^, which place primary emphasis on regular daily practice and include only brief periods (e.g., one day) of intensive retreat practice. The current study provides suggestive evidence that intensive retreat practice may deserve increased consideration by researchers as an “active ingredient” in mindfulness training. This may be especially relevant if we hope to go beyond studying mindfulness practice in a short-term intervention model, and investigate its public health impact as an ongoing, long-term health-related strategy for behavior change.

One limitation of the current findings is their ecological validity, since data were collected in a laboratory setting. Although respiration recordings were made outside of formal practice settings, it is also possible that LTMs spontaneously engaged in meditation practice during these periods. One way to address these limitations would be to assess RR across a variety of tasks, especially in naturalistic settings outside the laboratory. Data of this kind would constitute a valuable extension to the present work, by establishing that the observed association between practice experience and RR persists throughout everyday behavior. We hope that the increasing quality and availability of tools for naturalistic recording of physiological data, via mobile and wireless sensor technology, will facilitate this type of study in future. Moreover, the measures available for the current study were constrained by the larger study from which they are drawn, and allowed only for analysis of temporal aspects of the respiration signal. Many dimensions of respiratory function deserve further assessment in contemplative practitioners, including, for example, absolute volume, efficiency, abdominal-thoracic balance, and gas exchange measures.

In sum, the novel questions raised by the current study highlight the importance of a nuanced approach to contemplative research that accounts for how the effects of practice may vary according to the specifics of practice experience. More broadly, in developing a mechanistic understanding of mindfulness training, it will be important to distinguish the contributions and interactions of cognitive and physiological pathways for change. Given its prominent role in formal meditation practice, and centrality in the body’s physiological processes, continued attention to respiration should be an important priority in research on mindfulness training.

## Methods

### Participants and recruitment

The sample included 69 participants, comprised of 31 long-term meditators (LTM) and 38 meditation-naïve participants (Control). Groups were age- and gender-matched by mean (LTM: M = 50.7 years, range 28 to 62; Control: M = 47.9 years, range 25 to 65) and gender (LTM: 17 (55%) female; Control: 26 (68%) female).

Inclusion in the LTM group required at least 3 years of formal experience with mindfulness-related meditation practice; ongoing daily practice of 30 minutes or more; and completion of at least 3 intensive meditation retreats of five days or longer. Participants were screened for cardiovascular-related health issues such as smoking, alcohol dependence, chronic bronchitis, high blood pressure, diabetes, high cholesterol, pulmonary hypertension, coronary artery disease, heart attacks, brain damage, or seizures, and other mental disorders (anxiety, depression, bipolar, ADHD, schizophrenia/schizoaffective disorder). Exclusion criteria for both groups also included current psychiatric medication use, psychiatric diagnosis within the past year. Other exclusion criteria, specific to Controls, included experience with meditation or mind-body techniques, and extensive exercise (more than 5x per week in an active sport or other recreational activity). LTMs were recruited via meditation centers across the United States and related mailing lists, as well as flyers and newspaper advertisements. Meditation-naïve participants were recruited from the Madison, WI area via flyers, online ads, and local media.

The data analyzed here were collected as part of a larger study, as described in previous publications by our group^22^,^23^,^30^. Informed consent was obtained from all participants, and monetary compensation was provided for participation. All study protocols were approved by the UW-Madison Health Sciences Institutional Review Board, and procedures were carried out in accordance with the approved guidelines.

### Experimental sessions

Participants visited the lab on three occasions, spaced on average 4.5 months apart (M = 135 days, range 70 to 266). Each session consisted of a 24-hour lab visit, including a standardized battery of behavioral, physiological and neuroimaging and questionnaire measures. Immediately prior to the second and third sessions, LTM participants completed an 8-hour day of formal practice, consisting of either Open Monitoring or Loving Kindness meditation counterbalanced across the two sessions. A subset of Control participants (n = 18) participated in a day of leisure activities immediately prior to the third session. The day of leisure for the Control group was designed to match the days of practice for the LTMs. The sitting sessions were comprised of activities such as reading, playing computer games and watching documentaries. No social interaction, which included the use of internet (email) or cell phones, took place between subjects for either group. The administration of food and drink was held constant for both groups. All data collection occurred after the completion of the day of practice. Further details of the day of practice and day of leisure protocols are available in previous publications by our group^25^.

### Practice history

Participants in the LTM group completed a structured practice history detailing types of meditation practice, durations, and practice setting (intensive retreat, or routine daily practice). For LTMs, cumulative lifetime practice hours were estimated separately for intensive retreats (M = 4,658, range 258 to 29,710) and daily practice (M = 4,495, range 954 to 14,172), and combined to determine total lifetime hours of practice (M = 9,081, range 1,439 to 32,612). Percentage of total hours on retreat varied from 2% to 91% (M = 40%). Prior to analysis, all variables representing practice hours were log-transformed to normalize their distribution. Retreat and Daily practice hours were not significantly correlated either before log transformation, r(29) = −0.17, p = 0.37, or afterwards, r(29) = −0.09, p = 0.63.

In their meditation history interviews, LTM participants estimated percentages of time spent on specific practices, according to the categories of Open Monitoring (OM), Focused Attention (FA) or Loving Kindness (LK)^29^,^31^. Open Monitoring (OM) describes practice that emphasizes maintaining non-judgmental awareness of the spontaneous flow of thought, emotion and sensation. Focused Awareness (FA) practices emphasize maintaining concentrated attention on a single object of experience, such as the sensation of breathing. Loving Kindness (LK), here short for loving-kindness and compassion meditation, is a specialized category of concentration practice in which emphasis is placed on cultivating these mental states, particularly through wishing well-being towards self and others. These practice types accounted for, on average, 97% of formal practice hours (range 75–100%). OM and FA accounted for, on average, 84% of total practice hours (range 56–100%), and 84% of retreat hours (range 46–100%). After log-transformation, total hours of practice, and hours for the core practice categories (OM + FA), were extremely highly correlated, r = 0.98. Given the predominance of these two practice types, we did not attempt to statistically distinguish between specific forms of practice in the LTM group, and we rely on total hours of formal sitting meditation as our primary measure of mindfulness training.

### Respiration measures

At each study session, inductance plethysmography was used to capture respiration signal during a seated, eyes-open, uninstructed resting period lasting 370 seconds. Respiration signal was recorded on a polygraphic input box developed by Electrical Geodesics, Inc., using an abdominally placed ProTech DuraBelt #1073622 belt.

From each recording session, we calculated mean respiration rate (RR) using time-domain analysis, following established guidelines^32^,^33^. Processing of the recorded signal was performed using Matlab r2014b (The MathWorks Inc., Natick, MA). Segments with distorted or missing signal were excluded from further processing; recordings without any detectable signal were excluded from further analysis. Usable data was available for 30 LTM and 35 Controls at the Baseline session; 24 LTM for the Open Monitoring practice session, alongside 30 Control participants; and 21 LTM for the Loving-Kindness practice session, alongside 29 Control participants. Groups remained matched on age and gender after exclusion.

Within a recording, peaks and troughs were identified as those local minima and maxima with amplitude exceeding (root mean square) relative to the centered signal, and separated by a minimum duration of 1 second. Each recording was inspected visually to confirm that intervals represented valid respiration cycles. RR was calculated as the mean trough-to-trough interval for all valid cycles, in units of breaths per minute (b/m). Volume and efficiency (volume/time) measures were not calculated, as accurate between-subjects comparison would require spirometric calibration procedures that were not available from the study design^32^.

### Statistical analysis

Analyses were conducted in R (3.1.1, R Foundation for Statistical Computing, http://www.r-project.org/). Mixed effects models were fit using the *lme4* package (1.1). Results of mixed-effects models were consistent with ordinary least-squares (OLS) regressions on weighted average and session-by-session RR values. In all mixed-effects models, a random effect of Participant was included, with intercept estimated at the first level. All other predictors were modeled as fixed factors. Effect sizes are reported in observed units (e.g. breaths/min). Maximum likelihood t- and F-tests were conducted using Satterthwaite approximations for pooled degrees of freedom, using the *lmerTest* package (2.0), with approximated degrees of freedom reported to nearest integer values. Significance tests for post-hoc contrasts used Tukey’s HSD correction for multiple comparisons.

## Additional Information

**How to cite this article**: Wielgosz, J. *et al.* Long-term mindfulness training is associated with reliable differences in resting respiration rate. *Sci. Rep.* **6**, 27533; doi: 10.1038/srep27533 (2016).
